# Is glucose-6-phosphatase dehydrogenase deficiency associated with severe outcomes in hospitalized COVID-19 patients?

**DOI:** 10.1038/s41598-021-98712-3

**Published:** 2021-09-28

**Authors:** Nitya Kumar, AbdulKarim AbdulRahman, Abdulla Ismaeel AlAwadhi, Manaf AlQahtani

**Affiliations:** 1 Royal College of Surgeons in Ireland, Busaiteen, Bahrain; 2 Mohammed Bin Khalifa Cardiac Centre, Manama, Bahrain; 3 Bahrain Defense Force Hospital - Royal Medical Services, Riffa, Bahrain

**Keywords:** Epidemiology, Outcomes research, Respiratory signs and symptoms, Risk factors, Viral infection

## Abstract

Glucose-6-phosphate dehydrogenase deficiency (G6PDd) is known to suppress the antioxidant system and is likely to aggravate severity of COVID-19, which results in a pro-oxidant response. This possible association has not been explored adequately in human studies. In this research, we report that the occurrence of non-invasive ventilation, intubation or death—all of which are indicative of severe COVID-19, are not significantly different in hospitalized COVID-19 patients with and without G6PDd (4.6 vs. 6.4%, *p* = 0.33). The likelihood of developing any of these severe outcomes were slightly lower in patients with G6PDd after accounting for age, nationality, presence of comorbidities and drug interventions (Odds ratio 0.40, 95% confidence intervals 0.142, 1.148). Further investigation that extends to both, hospitalized and non-hospitalized COVID-19 patients, is warranted to study this potential association.

## Introduction

Glucose-6-phosphate dehydrogenase deficiency (G6PDd) is the most common enzyme deficiency in humans, affecting about 400 million people worldwide, with a high prevalence in persons of African, Asian, and Mediterranean descent^1^. An X linked disorder, G6PDd is caused by deficiency of Glucose-6-phosphate dehydrogenase, a cytosolic enzyme that catalyzes the pentose phosphate shunt. This is required to maintain the level of NADPH, which protects the red blood cells from oxidative injury. Affected patients are usually asymptomatic, but can develop episodic hemolysis following exposure to drug, food or chemicals which can potentiate an oxidative stress on the red blood cells^2^. G6PDd has been hypothesized to exacerbate severity of SARS-CoV-2 infections, as, the virus prompts a pro-oxidant response while simultaneously suppressing the antioxidant system^3^. Given that G6PDd results in a compromised antioxidant system, it is postulated that these individuals might suffer more severely from COVID-19^4^. In addition, ex vivo studies show increased vulnerability of G6PD-deficient cells to SARS-CoV-2 infection compared to cells with healthy levels of G6PD^5^. The most recent observational research involving 182 COVID-19 patients in Sardinia, reported that hospitalized patients had a higher proportion of G6PDd compared those with asymptomatic or mild disease^6^. There is lack of adequate investigation on G6PDd and severity of COVID-19 clinical outcomes, specifically, whether G6PDd is associated with more severe disease outcomes in humans.. Bahrain has a high prevalence of G6PDd, estimated about 19 to 26.4%^7–9^. Moreover, Bahrain has recorded more than 120,000 COVID-19 cases up to date^10^. In light of these, the present study aimed to investigate whether G6PDd is associated with severe outcomes in hospitalized COVID-19 patients.

## Results

During 24th of February 2020 until 31st of July 2020, a random cohort of 1792 patients was sampled from the COVID-19 patients hospitalized at the participating treatment facilities and enrolled for this study. Out of these, 175 had G6PDd (9.76%). The demographic characteristics and pre-existing comorbidities for patients with and without G6PDd are shown in Table 1. Age distribution was comparable between the two groups; mean age of G6PDd patients being 46.9 ± 16.6 years and that for non-G6PDd being 45.8 ± 17.0 years. Males constituted 53.7% of the G6PDd group compared to 59.6% in the non-G6PDd category (*p* = 0.14). Diabetes Mellitus, Cardiovascular Disease (CVD), Chronic Obstructive Pulmonary Disease (COPD) and other Chronic Lung Diseases excluding Asthma and COPD were present to a comparable proportion across patients with and without G6PDd. Patients with G6PDd were predominantly Bahraini nationals (91.4% vs. 52.9%) and had higher prevalence of Sickle Cell Disease (9.7% vs, 1.3%), Hypertension (36.6% vs. 29%), Asthma (7.4% vs. 4.1%) and Chronic Kidney Disease (9.7% vs, 3.8%).

**Table 1:** Demographic characteristics and comorbidities in the study subjects.

Characteristics	G6PDd present (n = 175)	G6PDd absent (n = 1617)	*p*-value
N (%)	175 (9.8%)	1617 (90.2%)	
Age: (mean ± SD)	46.9 ± 16.6	45.8 ± 17.0	0.42
Male	94 (53.7%)	963 (59.6%)	0.14
Bahraini nationality	160 (91.4%)	855 (52.9%)	< 0.001
Sickle Cell Disease	17 (9.7%)	21 (1.3%)	< 0.001
Diabetes Mellitus	48 (27.4%)	469 (29.0%)	0.66
Cardiovascular Disease (CVD)	24 (13.7%)	156 (9.6%)	0.089
Hypertension	64 (36.6%)	469 (29.0%)	0.038
Asthma	13 (7.4%)	67 (4.1%)	0.046
Chronic Obstructive Pulmonary Disease (COPD)	0 (0.0%)	7 (0.4%)	0.38
Chronic Kidney Disease (CKD)	17 (9.7%)	62 (3.8%)	< 0.001
Other Chronic Lung Disease (Not asthma nor COPD)	9 (0.6%)	9 (0.6%)	0.32

The presenting symptomatology in the study subjects was quite varied and has been summarized in Table 2. Almost two thirds of the patients showed some symptoms at admission in both G6PDd and non-G6PDd groups (59.4% and 63.8% respectively), and cough was found to be the most common symptom in both groups (41.1% and 42.1% respectively). It can be seen that both G6PDd and non-G6PDd patients were comparable with respect to the symptoms on admission.

**Table 2:** Findings upon admission in the study subjects.

Findings	G6PDd present (n = 175)	G6PDd absent (n = 1617)	*p*-value
Symptomatic on admission:	104 (59.4%)	1031 (63.8%)	0.26
Fever (> 38C)	44 (25.1%)	357 (22.1%)	0.36
Cough	72 (41.1%)	681 (42.1%)	0.80
Chest pain	15 (8.6%)	144 (8.9%)	0.88
Shortness of breath	29 (16.6%)	331 (20.5%)	0.22
Loss of smell	9 (5.1%)	47 (2.9%)	0.11
Loss of taste	6 (3.4%)	48 (3.0%)	0.74
Diarrhea	10 (5.7%)	94 (5.8%)	0.96
Nausea or vomiting	10 (5.7%)	83 (5.1%)	0.74
Body pain	25 (14.3%)	253 (15.6%)	0.64
Heart rate on admission, beats per minute (mean ± SD)	88.3 ± 12.7	86.5 ± 14.4	0.11
SBP on admission: mmHg (mean ± SD)	131.8 ± 19.4	130.2 ± 18.6	0.30
DBP on admission: mmHg (mean ± SD)	77.6 ± 13.0	77.1 ± 11.6	0.64
Respiratory rate on admission: breath per minute (mean ± SD)	19.6 ± 4.6	20.1 ± 7.1	0.69
Elevated ALT (> 40U/L)	34 (20.5%)	374 (25.1%)	0.19
Elevated creatinine	21 (12.0%)	148 (9.2%)	0.22
**Chest radiographic findings**			
Pneumonia	51 (31.9%)	480 (35.0%)	0.43
Normal	109 (68.1%)	892 (65.0%)	
Hypotension (SBP < 90 mmHg or DBP < 60 mmHg) on admission	12 (6.9%)	65 (4.0%)	0.079
Tachypnea (RR > 22) on admission	4 (2.3%)	45 (2.8%)	0.70
**Disease severity on admission**			
Admitted on room Air	155 (88.6%)	1420 (87.9%)	0.75
Admitted on oxygen support	20 (11.4%)	185 (11.4%)	
Admitted on NIV	0 (0.0%)	8 (0.5%)	
Mechanical ventilation	0 (0.0%)	3 (0.2%)	
Oxygenation on admission	20 (11.4%)	196 (12.1%)	0.79

*ALT* alanine aminotransferase, *NIV* non-invasive ventilation.

The drug interventions received by the subjects (Table 3) included Azithromycin, Hydroxychloroquine, Ribavirin, Kaletra, Tocilizumab and convalescent plasma. The number of G6PDd and non-G6PDd patients who received these were similar for most treatments except for azithromycin and hydroxychloroquine. Azithromycin was received by higher number of people with G6PDd (33.1% vs. 23.9%). Hydroxychloroquine was used more for people without G6PDd (31.2% vs. 18.9%).

**Table 3:** Drug interventions during hospital stay.

Medication	G6PDd present (n = 175)	G6PDd absent (n = 1617)	*p*-value
Hydroxychloroquine	33 (18.9%)	504 (31.2%)	< 0.001
Azithromycin	58 (33.1%)	387 (23.9%)	0.007
Kaletra	31 (17.7%)	293 (18.1%)	0.89
Ribavirin	27 (15.4%)	246 (15.2%)	0.94
Steroids	20 (11.4%)	184 (11.4%)	0.98
Tocilizumab	8 (4.6%)	74 (4.6%)	1.00
Convalescent plasma transfusion	7 (4.0%)	61 (3.8%)	0.88

Main outcome in the study included requirement of non-invasive ventilation or mechanical ventilation or death. The proportion of patients who suffered either of these outcomes was similar in G6PDd and non-G6PDd patients and ranged between 2.3 and 3.4% as described in Table 4.

**Table 4:** Occurrence of primary outcome in G6PDd and non-G6PDd patients.

Outcome	G6PDd present (n = 175)	G6PDd absent (n = 1617)	*p*-value
Primary outcome (requirement of either NIV or mechanical ventilation or death)	8 (4.6%)	104 (6.4%)	0.33
NIV	4 (2.3%)	53 (3.3%)	0.48
Mechanical ventilation	4 (2.3%)	44 (2.7%)	0.73
Death	4 (2.3%)	55 (3.4%)	0.43

*NIV* non-invasive ventilation.

The crude odds ratio of suffering the primary outcome for G6PDd patients was found to be 0.69 (95% CI 0.33, 1.45), as shown in Table 5. After adjusting for age, nationality, comorbidities (HTN, CKD, COPD, SCD) and drug interventions, the OR decreased slightly to 0.403 (0.142, 1.148), but remained statistically non-significant (*p* = 0.132, Supplementary Table S1). We also evaluated whether the co-presence of G6PDd and SCD had differential effect on the outcome, by testing for effect measure modification (Supplementary Table S2). SCD and G6PD did not appear to modify each other’s effect on the primary outcome (*p* = 0.92).

**Table 5:** Odds of suffering the primary outcome in patients with G6PDd compared to non-G6PDd patients.

Type of analysis	OR (95% CI)	*p* value
Crude	0.69 (0.33, 1.45)	0.337
Adjusted for age, nationality, drug interventions, comorbidities (HTN, COPD, CKD, SCD)	0.403 (0.142, 1.148)	0.089

*HTN* Hypertension, *COPD* Chronic Obstructive Pulmonary Disease, *CKD* Chronic Kidney Disease, *SCD* Sickle Cell Disease.

## Discussion

The present study did not find hospitalized patients with G6PDd to have higher odds of requiring NIV, HFNC, MIV or death, after accounting for age, nationality, drug interventions and comorbidities (HTN, COPD, CKD SCD).

The patients in both G6PDd and non-G6PDd group were comparable in terms of age and sex. Our cohort was considerably younger with a mean age 45.9 years, compared to hospitalized patients in United States^11,12^ (median age 65 years) and United Kingdom^13^ (mean age of 70.5 years). The G6PDd groups was predominantly Bahraini nationals as compared to other nationalities, which is not surprising given that the prevalence of G6PDd has been reported to be highly prevalent in the native population of Bahrain from 19 to 26.4%^7–9^ and to the extent of 47% in those with sickle cell trait^8^. Owing to the same phenomenon, the occurrence of SCD was significantly high in the G6PDd group (9.7%) compared to the non-G6PD group (1.3%). Given recent studies on SCD and risk of severe COVID-19 outcomes^14–16^, we evaluated whether the copresence of SCD and G6PD would affect the outcome by looking at presence of effect measure modification of SCD on the G6PDd and the primary outcome. The result showed that SCD didn’t alter the risk of the primary outcome in G6PDd patients (Supplementary Table S2).

The medications used in this cohort should be interpreted with the study timeline. Early in the pandemic HCQ and Azithromycin received wide attention in its use in COVID19 patients^17^. Similarly, Kaletra and Ribavirin in were also used^18–20^. Hence, it was common to prescribe these drugs in hospital settings at that time. Moreover, the use of antibiotics was common in patients with pneumonia. As more evidence about the lack of benefit from these drugs and the potential harm was released, the use of these medications has decreased^21–26^. The benefit of steroids in hypoxic COVID19 patients was reported in June^26^ and it was only after that that steroids were used in hospitalized patients.

HCQ is known to increase risk of acute hemolysis in G6PDd patients and hence it is relatively contraindicated in G6PDd patients. This have led to the reduced use of HCQ in G6PDd patients. On the other hand, Azithromycin was used more commonly in G6PDd patients as the use of HCQ was limited. This has caused more patients in the G6PDd group to receive Azithromycin. The combination between the two drugs have been linked to increased risk of arrythmia, QT prolongation and this could explain the lower use of combination between HCQ and Azithromycin in the non G6PDd patients^27^.

G6PDd group was relatively worse compared to non-G6PDd counterparts with respect to presence of HTN, Asthma and CKD. Meta-analyses have reported HTN to confer a two-fold increase in hazards of mortality (adjusted HR 2.12; 95% CI 1.17, 3.82)^28,29^, while asthma being a respiratory condition is highly associated with worse outcomes in COVID-19 patients with an RR of 1.96; 95% CI 0.89, 4.33^30^. In addition, CKD has been reported to result doubling of the risk of mortality (RR: 2.11; 95% CI 1.72, 2.58) in hospitalized patients^30^. The higher prevalence of these conditions in G6PDd patients kept them at a higher baseline risk to develop worse disease outcomes.

The occurrence of CVD, DM and other Chronic Lung Diseases was not affected by G6PDd status. Diabetes has been established to be a one of the key risk factors severe COVID-19 outcomes and is significantly associated with death or tracheal intubation with 7 days of hospitalization^31^. Prospective studies have reported a two-fold increase in odds of death with CVD (OR 2.46; 95% CI 0.755, 8.044)^28^. Since both these major comorbidities were equally present in both patients with and without G6PD, it is likely that the risk of outcome attributable to these comorbidities would have been apportioned between the groups.

Patients had similar profile and range of presenting symptoms across the G6PDd and non-G6PDd groups with no significant differences. However, it is worth noting that the non-G6PDd group had marginally higher occurrence of symptoms (63.8%) compared to G6PD (59.4%). In addition, relatively larger proportion of the non-G6PDd required mechanical ventilation, non-invasive ventilation as well as oxygenation upon admission and hence, seemed to be worse off compared to the G6PDd group. One of the reasons for this could be that the G6PDd patients could have had a lower admission threshold because of presence of G6PDd and were perceived to be at a higher risk. This could be especially true given G6PDd deficient patients had significantly higher proportion of high-risk comorbidities of SCD, HTN, Asthma and CKD, as well as marginally higher occurrence of CVD.

Initially during the pandemic in Bahrain, patients with risk factors were admitted to hospitals even if they had mild disease. As opposed to cases without risk factors, who were hospitalized based on a clinical decision. Risk factors for severe COVID 19 included: Older Age, Smoking, Obesity, DM, CVD, Respiratory diseases and lung cancers^32–34^.

Patients in the G6PDd group were older on baseline and had more of these risk factors, namely CVD HTN and CKD. This has led to a lower hospitalization threshold for the G6PDd patients. This is also reflected upon the severity of disease in both groups. The G6PDd group were less symptomatic and none required advanced oxygenation on admission to the hospital, as most were admitted early during their disease course. Therefore, more patients with G6PDd were hospitalized when compared to non G6PDd patients. Patients who were in home isolation were not studied in this research and hence the outcomes of G6PDd on a population level could not be measured. Owing to these reasons, milder cases of COVID-19 in G6PDd were disproportionately high in this cohort. This has the potential to bias the results towards the null.

One of the main strengths of this study lies in the inclusion of majority of the national tertiary care hospitals that provided care for hospitalized COVID-19 patients. The fact that data collection from the EMR was done manually, reviewed and checked by the study authors and contributors, speaks to the quality of the data. The present study represents the only available research in Bahrain that looks at the role of G6PDd and COVID-19 outcomes.

Our study is restricted to hospitalized COVID-19 patients and therefore it is not possible to generalize the results to COVID-19 cases in the outpatient setting. Due to the same reason, it is not possible to infer the effect of G6PDd on COVID-19 symptomatology in milder cases that did not require hospitalization. Given the data was obtained from EMR, it is possible that the lifestyle factors not captured in EMR such as smoking and obesity, could not be accounted for in the analysis and could be potential confounders. In addition, time from symptom onset, inflammatory markers and frequency of G6PDd related admissions patients were also not captured in the EMR and could be source of unmeasured confounding.

In conclusion, the present study did not find any association between presence of G6PDd and occurrence of serious outcomes in COVID-19 patients. We recommend further large-scale studies that include both hospitalized and non-hospitalized COVID-19 patients, for a more comprehensive insight on the role of G6PDd the pathophysiology of COVID-19 outcomes**.**

## Methods

### Study population, patient selection and data extraction

The study population consisted of COVID-19 cases that were admitted at Bahrain Ministry of Health COVID-19 treatment facilities between 24 February to 31 July 2020. The four participating COVID-19 treatment facilities included Ebrahim bin Khalil Kanoo COVID-19 Centre, Salmaniya Medical Complex COVID-19 Centre, Hereditary Blood Disorder Centre (HBDC) COVID-19 Centre and Jidhafs COVID-19 Centre. Data on these patients was obtained from the electronic medical record (EMR) system, “I-SEHA”, which contains patient records and clinical information as text files. Patients with and without G6PDd were sampled randomly. Patients were included if they were above 18 years of age, had a diagnosis of COVID-19 disease and were admitted between the above specified dates. Data was extracted manually from EMR by five physicians and ten senior medical students, who reviewed all the cases and filled an electronic patient record form developed to collect data for this study.

### Diagnosis of COVID-19

All patients were confirmed to be infected by SARS-CoV-2 by a polymerase chain reaction (PCR) test of a nasopharyngeal sample. The PCR test was conducted using Thermo Fisher Scientific (Waltham, MA) TaqPath 1-Step RT-qPCR Master Mix, CG on the Applied Biosystems (Foster City, CA) 7500 Fast Dx RealTime PCR Instrument. The assay used and targeted the E gene. If the E gene was detected, the sample was then confirmed by RdRP and N genes. The E gene Ct value was reported and used in this study. Ct values > 40 were considered negative. Positive and negative controls were included for quality control purposes.

### Exposure assessment

The exposure of interest was presence of G6PDd, information on which was obtained from the medical records. The original diagnosis of G6PDd was made through fluorescence spot test using whole blood. Patients with intermediate levels of G6PD were not considered as having the exposure of interest.

### Outcome assessment

Primary outcome in this study was a composite endpoint that included either requirement of non-invasive ventilation, intubation or death.

### Covariates

Information was collected on patients’ demographic details, vital signs, laboratory test results, medication lists, past medical history, clinical severity scale (as seen in the Supplementary Table attached in the appendix), oxygenation requirement on admission, the ratio of the oxygen saturation to the fraction of inspired oxygen (SpO2:FiO2) at admission, requirement of ICU care, ventilator use and outcomes. A complete list of variables collected is attached in Appendix A.

### Statistical analysis

Categorical variables have been described using frequencies and percentages and continuous variables, as means and standard deviations. Bivariate associations between the G6PDd status groups and the measured patient characteristics have been analyzed using Chi-squared (χ2) tests for categorical variables and t-test for continuous variables. A multi-variable logistic regression model was used to estimate the association between G6PDd and the primary and secondary outcomes. The primary outcome model adjusted for demographic factors, clinical factors and medications. Effect modification was analyzed using interaction term in a regression model. All analyses were performed using STATA software, version 16, (StataCorp. 2019. Stata Statistical Software: Release 16. College Station, TX: StataCorp LLC.).

### Ethics approval and consent to participate

The protocol and manuscript for this study were reviewed and approved by the National COVID-19 Research Committee in Bahrain (Approval Code: CRT-COVID2020-061). This committee has been jointly established by the Ministry of Health and Bahrain Defense Force Hospital research committees in response to the pandemic, to facilitate and monitor COVID-19 research in Bahrain. All methods and retrospective analysis of data was approved by the National COVID-19 Research and Ethics Committee and carried out in accordance with the local guideline and ethical guidelines of the Declaration of Helsinki 1975. All data used in this study was collected as part of normal medical procedures. Informed consent was waived by the National COVID-19 Research and Ethics Committee for this study due to its retrospective and observational nature and the absence of any patient identifying information.

## Supplementary Information

Supplementary Information.

## Data Availability

Original data will be made available upon request to the corresponding author.
