# Supplementary material for: Is glucose-6-phosphatase dehydrogenase deficiency associated with severe outcomes in hospitalized COVID-19 patients?
Source: Sci Rep. 2021 Sep 28;11:19213. doi: 10.1038/s41598-021-98712-3 (PMC8478975; doi:10.1038/s41598-021-98712-3)
Supplement: Supplementary file 1 — Supplementary Information. [file 41598_2021_98712_MOESM1_ESM.pdf]

# Supplementary Tables

**Title:** Is glucose-6-phosphatase dehydrogenase deficiency associated with severe outcomes in hospitalized COVID-19 patients?

**Authors:**

Nitya Kumar [1]

AbdulKarim AbdulRahman [2]

Abdulla Ismaeel AlAwadhi [2]

Manaf AlQahtani [1]

1. Royal College of Surgeons in Ireland, Bahrain

2. Mohammed Bin Khalifa Cardiac Centre, Bahrain

## S1: STATA Output for logistic regression model

**Logistic regression**

| outcome | Coef. | St.Err. | t-value | p-value | [95% Conf | Interval] | Sig |
|---|---|---|---|---|---|---|---|
| G6PD | .403 | .215 | -1.70 | .089 | .142 | 1.148 | * |
| Age | 1.002 | .012 | 0.18 | .856 | .979 | 1.026 | |
| Nationality | .582 | .19 | -1.66 | .097 | .307 | 1.103 | * |
| HTN | 2.981 | 1.075 | 3.03 | .002 | 1.47 | 6.045 | *** |
| COPD | 13.432 | 20.272 | 1.72 | .085 | .697 | 258.697 | * |
| CKD | 8.916 | 3.606 | 5.41 | 0 | 4.036 | 19.697 | *** |
| Azithromycin | 2.528 | .773 | 3.03 | .002 | 1.388 | 4.604 | *** |
| Ribavirin | 3.8 | 1.172 | 4.33 | 0 | 2.076 | 6.955 | *** |
| Steroids | 5.991 | 1.782 | 6.02 | 0 | 3.345 | 10.731 | *** |
| Plasma | 8.515 | 3.608 | 5.06 | 0 | 3.711 | 19.536 | *** |
| HCQ | .404 | .144 | -2.54 | .011 | .201 | .812 | ** |
| Tocilizumab | 21.118 | 7.816 | 8.24 | 0 | 10.223 | 43.621 | *** |
| Constant | .006 | .004 | -8.68 | 0 | .002 | .02 | *** |

| | | | |
|---|---|---|---|
| Mean dependent var | 0.063 | SD dependent var | 0.242 |
| Pseudo r-squared | 0.534 | Number of obs | 1792.000 |
| Chi-square | 447.606 | Prob > chi2 | 0.000 |
| Akaike crit. (AIC) | 416.303 | Bayesian crit. (BIC) | 487.687 |

**** p<.01, ** p<.05, * p<.1*

**Abbreviations: HTN: Hypertension, COPD: Chronic Obstructive Pulmonary Disease, CKD: Chronic Kidney Disease, HCQ: Hydroxychloroquine**

## S2: Interaction between G6PDd and SCD

**Logistic regression**

| outcome | Coef. | St.Err. | t-value | p-value | [95% Conf | Interval] | Sig |
|---|---|---|---|---|---|---|---|
| G6PD | .679 | .272 | -0.97 | .333 | .31 | 1.487 | |
| SCD | 1.542 | 1.157 | 0.58 | .564 | .354 | 6.711 | |
| G6PDxSCD | .874 | 1.165 | -0.10 | .92 | .064 | 11.907 | |
| Constant | .068 | .007 | -26.23 | 0 | .056 | .083 | *** |

| | | | |
|---|---|---|---|
| Mean dependent var | 0.063 | SD dependent var | 0.242 |
| Pseudo r-squared | 0.002 | Number of obs | 1792.000 |
| Chi-square | 1.380 | Prob > chi2 | 0.710 |
| Akaike crit. (AIC) | 844.529 | Bayesian crit. (BIC) | 866.494 |

**** p<.01, ** p<.05, * p<.1*

**Abbreviations: SCD: Sickle Cell Disease**
